# Inhibition of breast cancer xenografts in a mouse model and the induction of apoptosis in multiple breast cancer cell lines by lactoferricin B peptide

**DOI:** 10.1111/jcmm.16748

**Published:** 2021-07-08

**Authors:** Rizdwan Rahman, Alyssa Danielle Fonseka, Shiang‐Chia Sua, Munirah Ahmad, Ramkumar Rajendran, Stephen Ambu, Fabian Davamani, Alan Soo‐Beng Khoo, Ebenezer Chitra

**Affiliations:** ^1^ School of Post Graduate Studies International Medical University Kuala Lumpur Malaysia; ^2^ School of Health Sciences International Medical University Kuala Lumpur Malaysia; ^3^ School of Medicine International Medical University Kuala Lumpur Malaysia; ^4^ Molecular Pathology Unit Cancer Research Centre Institute for Medical Research National Institutes of Health Ministry of Health Malaysia Shah Alam, Selangor Malaysia; ^5^ School of Pharmacy International Medical University Kuala Lumpur Malaysia; ^6^ Institute for Research Development and Innovation International Medical University Kuala Lumpur Malaysia

**Keywords:** anticancer peptide, breast cancer, lactoferricin B, milk peptide, natural peptide

## Abstract

Breast cancer has a diverse aetiology characterized by the heterogeneous expression of hormone receptors and signalling molecules, resulting in varied sensitivity to chemotherapy. The adverse side effects of chemotherapy coupled with the development of drug resistance have prompted the exploration of natural products to combat cancer. Lactoferricin B (LfcinB) is a natural peptide derived from bovine lactoferrin that exhibits anticancer properties. LfcinB was evaluated in vitro for its inhibitory effects on cell lines representing different categories of breast cancer and in vivo for its suppressive effects on tumour xenografts in NOD‐SCID mice. The different breast cancer cell lines exhibited varied levels of sensitivity to apoptosis induced by LfcinB in the order of SKBR3>MDA‐MB‐231>MDA‐MB‐468>MCF7, while the normal breast epithelial cells MCF‐10A were not sensitive to LfcinB. The peptide also inhibited the invasion of the MDA‐MB‐231 and MDA‐MB‐468 cell lines. In the mouse xenograft model, intratumoural injections of LfcinB significantly reduced tumour growth rate and tumour size, as depicted by live imaging of the mice using in vivo imaging systems (IVIS). Harvested tumour volume and weight were significantly reduced by LfcinB treatment. LfcinB, therefore, is a promising and safe candidate that can be considered for the treatment of breast cancer.

## 1 INTRODUCTION

Breast cancer is one of the most common types of cancer worldwide, particularly among women, with incidence and mortality rates of 24.2% and 15%, respectively,^1^ despite being targeted by multipronged therapies. Breast cancer is diverse and heterogeneous with respect to the expression of genes, receptors and intracellular signalling proteins, thereby exhibiting a complex disease aetiology and varied response to therapy. Three major growth factor receptors implicated in breast cancer are progesterone receptor (PR), oestrogen receptor (ER) and human epidermal growth factor receptor 2 (Her2/neu). Based on inter‐tumour heterogeneity recognized by molecular characterization, breast cancers are categorized as follows: luminal A (ER^high^, Her2^low^), luminal B (ER^low^, Her2^low^), Her2‐enriched (Her2^+^, ER^‐^), basal‐like or triple‐negative (ER^‐^, PR^‐^, Her2^‐^) and claudin‐low (ER^‐^, Claudin^low^, E‐cadherin^low^) types, most of which are associated with a poor short‐term prognosis.^2^

Her2‐positive breast cancer is characterized by Her2 receptor overexpression and dependence on Her2 pathway for survival. The prognosis of Her2‐positive breast cancers improved dramatically following the use of monoclonal antibodies such as trastuzumab and kinase inhibitors for therapy.^3^ Luminal B type is more aggressive and has worse prognosis than luminal A type.^4^ Triple‐negative breast cancer (TNBC) is highly aggressive, heterogeneous and the most difficult type of breast cancer to treat, with a 10 year survival rate of 60%‐70%. TNBC responds only to chemotherapy due to the absence of hormone receptors.^5^, ^6^ Antibody‐drug conjugates, adjuvant and neo‐adjuvant therapies have significantly increased patient outcome although toxicity, relapses and drug resistance are also reported.^3^ Chemotherapy is often accompanied by the development of drug resistance facilitated by multiple resistance mechanisms leading to serious side effects. This has prompted research into the development of natural agents that might help overcome these issues. Many studies have focused on anticancer peptides to explore their potential to kill cancer cells, but most were found to be highly toxic.

Bovine lactoferrin is an iron‐binding glycoprotein present in cow milk with antimicrobial, anticancer, and immunomodulatory properties.^7^ Administration of lactoferrin in vivo is safe and well‐tolerated, and is effective against some cancers.^8^ Lactoferricin B (LfcinB) is a 25‐amino acid peptide released from lactoferrin by acid‐pepsin hydrolysis. Similar to other cationic antimicrobial peptides, LfcinB exhibits antibacterial and antifungal properties.^9^, ^10^ as well as anticancer properties. Interference with cell cycle, induction of apoptosis, inhibition of cell migration and immunomodulation are proposed to be the mechanisms of action of lactoferrin and its peptide derivatives against cancer cells.^8^ LfcinB and a core peptide derived from Lfcin have been found to induce apoptosis of leukaemic T cells, B lymphoma cells and a gastric cancer cell line.^10^, ^11^, ^12^, ^13^ LfcinB also exerted an inhibitory effect against neuroblastoma cells and B lymphoma cells both in vitro and in vivo.^15^, ^16^ In MDA‐MB‐435 cells, LfcinB‐induced DNA fragmentation was enhanced by C6 ceramide.^17^ Several peptides derived from LfcinB have also been shown to exert anticancer effects.^17^, ^18^, ^19^

While these reports confirm the anticancer effects of LfcinB, a focused exploration of the ability of LfcinB to inhibit diverse breast cancer subtypes has yet to be undertaken. The present study found that the inhibitory effects of LfcinB peptide on cell lines representing different categories of breast cancer were varied. LfcinB also had antitumorigenic effect in an in vivo xenograft model of breast cancer in immunodeficient mice.

## 2 MATERIALS AND METHODS

### 2.1 Cell lines and cell culture

The MDA‐MB‐231, MDA‐MB‐468, SKBR3 and MCF7 breast cancer cell lines, and the MCF‐10A human mammary gland cell line, were purchased from the American Type Culture Collection. MDA‐MB‐231‐GFP‐luc2 cells previously transduced with lentiviral GFP‐luc2 (kindly provided by Dr Marco Herold from Walter and Elisa Hall Institute of Medical Research, Australia) were used for the in vivo experiments. The cancer cell lines were cultured in Dulbecco's modified Eagle's medium (DMEM) supplemented with 10% foetal bovine serum (FBS) and 1% penicillin‐streptomycin (Thermo Fisher Scientific, Inc), and maintained in a humidified incubator at 37˚C with 5% CO_2_. The MCF‐10A cell line was cultured in DMEM/F12 supplemented with 5% horse serum, 0.02% epidermal growth factor, 0.05% hydrocortisone, 0.1% insulin, 1% non‐essential amino acids, 1% glutamine and 1% penicillin‐streptomycin and maintained at 37˚C with 5% CO_2_. All the components were purchased from Thermo Fisher Scientific, Inc.

### 2.2 Peptide

LfcinB peptide (FKCRRWQWRMKKLGAPSITCVRRAF) was synthesized by Mimotopes Pty. Ltd. The peptide was dissolved in incomplete DMEM to a working concentration of 1 mg/ml.

### 2.3 Apoptosis assay

The assay was carried out using the Annexin V‐FITC early apoptosis detection kit from Cell Signaling Technology, Inc., following the manufacturer's instructions. The cells were cultured in 6‐well plates and treated with various concentrations of LfcinB peptide for 48 h. The cells were detached using PBS containing 10 mM EDTA, stained and analysed using a flow cytometer (BD FACSCalibur; BD Biosciences).

### 2.4 Cell invasion assay

Boyden chambers (8 μM pore size) were pre‐coated with fibronectin and placed in 24‐well companion plates. In the upper chamber, 2 × 10^4^ cells were placed in serum‐free medium containing various concentrations of LfcinB peptide. Complete DMEM (600 µl) was placed in the lower chamber. After 12 h of incubation, the cells that had invaded into the fibronectin‐coated lower surface of the membrane were fixed with methanol, stained with 1% crystal violet and counted using Nikon Eclipse Ti‐S inverted microscope at 200X magnification.

### 2.5 Animals

Immunodeficient NOD‐SCID‐gamma (NSG) mice were bred and maintained in the SPF facility of the Institute of Medical Research (IMR). Female mice (8 weeks old, weighing 18‐25 g) were used in the present study. The mice were housed in groups of 5 animals per cage maintained in a barrier facility equipped with HEPA‐filtered racks. All the experiments were carried out following the guidelines and approval of the Animal Care and Use Committee (ACUC) of Ministry of Health (ACUC/KKM/02 9/2015) (Sept 2015) and Medical Research and Ethics Committee, Ministry of Health Malaysia, as well as the IMU Joint Committee on Research and Ethics of International Medical University (IMU R 142/2014[6/2014]) (June 2014) within the approval period of 2015‐2017.

### 2.6 Breast cancer xenografts

**FIGURE 1:**
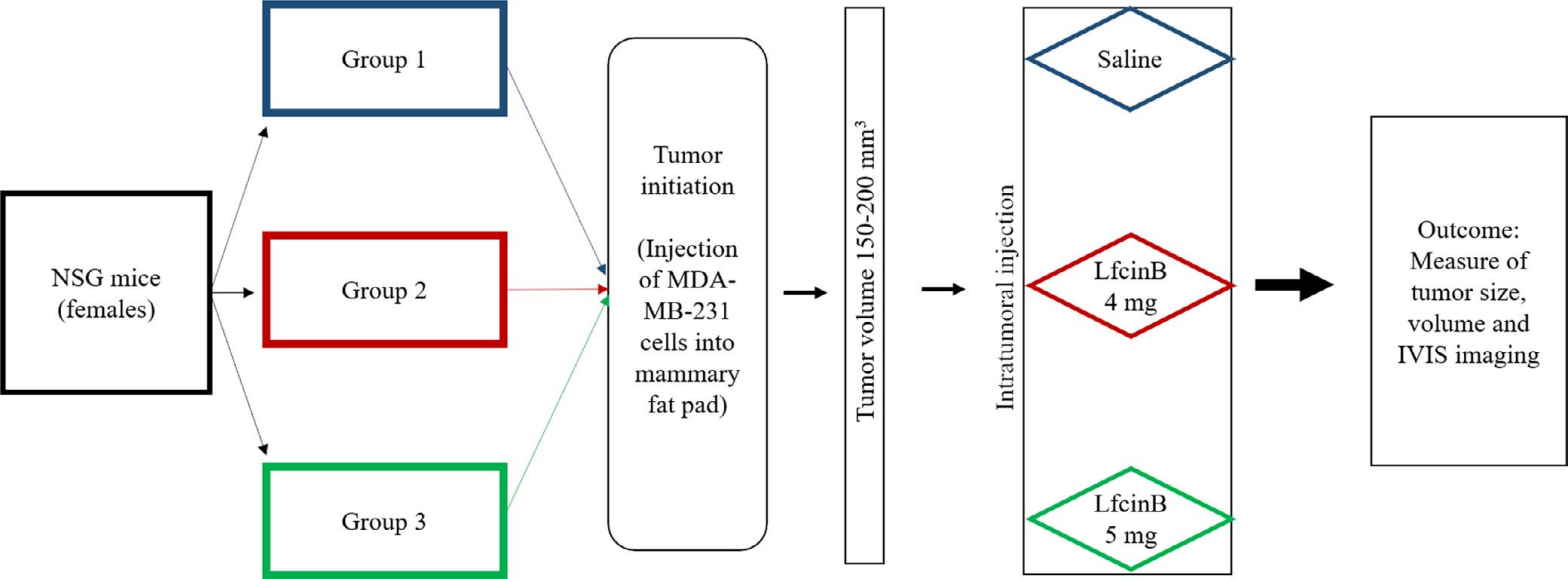
Flow chart depicting in vivo tumour induction and LfcinB treatment in NSG mice

MDA‐MB‐231‐GFP‐luc2 cells (1 × 10^6^ cells in 25 µl of plain DMEM mixed with 25 µl Matrigel (BD Biosciences)) were injected into the fifth left inguinal mammary fat pad of the female NSG mice. When the tumours became palpable, they were measured using callipers to assess tumour volume. When the tumour reached a volume of ~150‐200 mm^3^, an intratumoural injection of 50 µl of LfcinB peptide (4 or 5 mg) or saline was administered for 3 consecutive days. Once the tumours in the control group reached a volume of ~700 mm^3^ or a diameter of 11 mm, all mice were killed by an overdose of CO_2_ gas in a CO_2_ chamber (10%‐30% volume/min). The tumours were harvested and photographed, and their volume and weight were recorded. A flow chart of the in vivo experimental protocol is depicted in Figure 1.

### 2.7 In vivo imaging systems (IVIS)

For live imaging, the mice were anaesthetized by isoflurane (3% induction dose and 1.5%‐2% maintenance dose via a precision vaporizer) and injected with 100 µl of diluted XenoLight D‐Luciferin‐K^+^ salt, a bioluminescent substrate (PerkinElmer, Inc) intraperitoneally (i.p.) at a concentration of 150 mg/kg bodyweight. The mice were placed in the imaging chamber for fluorescent imaging using IVIS^®^ Spectrum (PerkinElmer, Inc) and imaged ventrally.

### 2.8 Histological analysis

The harvested tumours were fixed in 10% formalin and embedded in paraffin for histological analysis. The tissue blocks were cut into 4 µm sections using a microtome (RM2255; Leica Biosystems, Inc). Slides were prepared and stained with haematoxylin and eosin (H&E), and images were captured using Nikon Eclipse 80i microscope at 200X and 400X magnifications.

### 2.9 Statistical analysis

Data were analysed using a Student's t test. Tumour growth curves were compared using a one‐way analysis of variance (ANOVA) followed by post hoc Tukey's honest significant difference (HSD). Differences between treatments were considered significant at the *P* < .5 probability level.

## 3 RESULTS

### 3.1 Varied sensitivity of different breast cancer cell lines to LfcinB‐induced apoptosis

**FIGURE 2:**
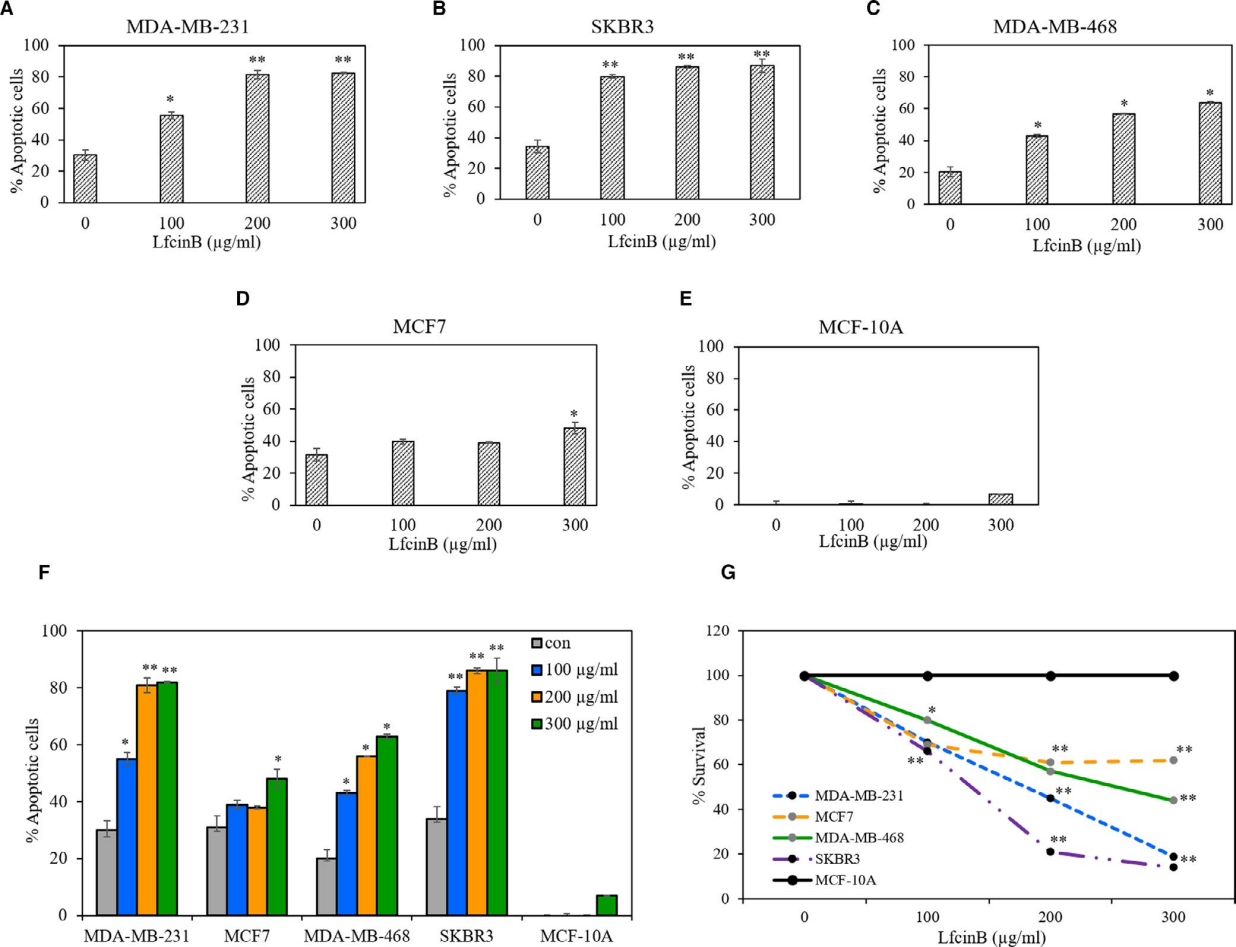
LfcinB induces apoptosis in different breast cancer cell lines. Percentage of apoptotic cells present in the breast cancer cell lines (A) MDA‐MB‐231 (B) SKBR3 (C) MDA‐MB‐468 (D) MCF7 and the normal breast cell line (E) MCF‐10A treated with 100‐300 µg/mL of LfcinB for 48 hours, stained with Annexin V‐FITC/PI and analysed by flow cytometry. Percentage of apoptotic cells include Annexin‐FITC/PI single positive as well as double‐positive cells. (F) Graph depicting the overall response of the different cell lines to LfcinB (G) Line graph depicting the comparative survival of the different cell lines treated with LfcinB, calculated from the apoptosis assay. The results are significant at *P < .05 or **P < .01

The different breast cancer cell lines exhibited varying degrees of sensitivity to LfcinB‐induced apoptosis, with the SKBR3 and MDA‐MB‐231 cells reaching ~80% cell death at the 100 and 200 µg/ml concentrations (Figure 2). In the MDA‐MB‐468 cells, LfcinB induced only ~60% cell death and in the MCF7 cells, only ~50% cell death at the 300 µg/ml concentration. The non‐tumorigenic breast epithelial cell line, MCF‐10A, exhibited only ~10% cell death, even at the 300 µg/ml concentration of LfcinB. Therefore, the SKBR3 and MDA‐MB‐231 cell lines were found to be highly sensitive to LfcinB, the MDA‐MB‐468 and MCF7 cells moderately sensitive and the MCF‐10A cells non‐responsive to LfcinB. The relative sensitivity of the different cell lines to LfcinB following treatment for 48 h is collectively depicted in Figure 2F for comparison. The survival percentage was calculated from the apoptosis assay and depicted as a line graph in Figure 2G to illustrate the relative survival of the different cell lines. Representative graphs from the flow cytometric analysis of apoptosis in the different cell lines using Annexin‐FITC and propidium iodide are depicted in Figure S1.

### 3.2 Inhibition of the invasion potential of breast cancer cell lines by LfcinB

**FIGURE 3:**
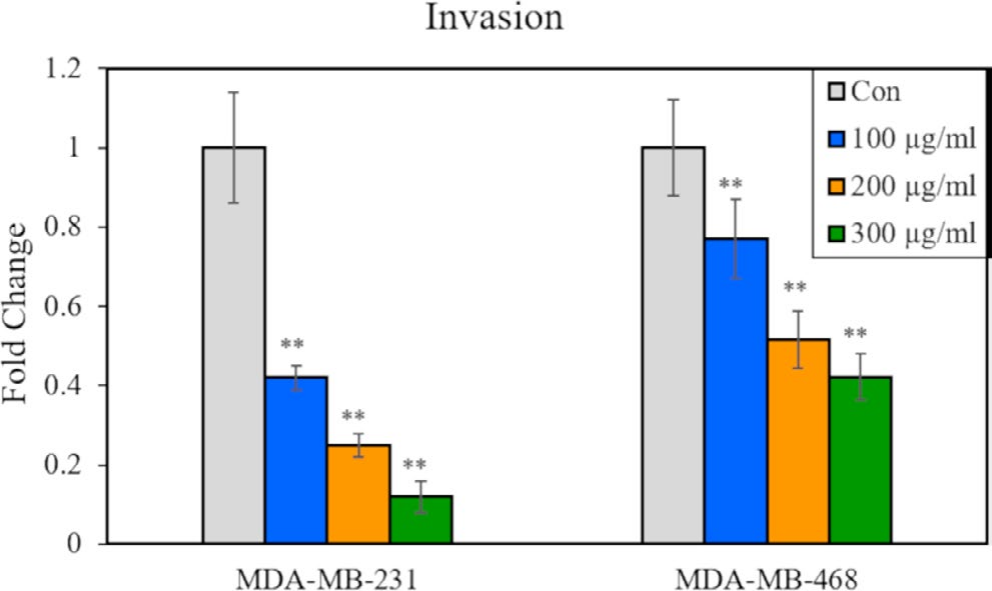
LfcinB inhibits invasion of breast cancer cell lines. Graph depicting the fold‐change in the relative number of invasive cells in MDA‐MB‐231 and MDA‐MB‐468 cell lines treated with 100‐300 µg/mL of LfcinB for 12 h as determined by Boyden chamber invasion assay. **The results are significant at *P* < .01

LfcinB inhibited the invasive potential of the highly invasive cell lines MDA‐MB‐231 and MDA‐MB‐468 in the Boyden chamber assay. The MDA‐MB‐231 cells treated with 200 µg/ml of LfcinB exhibited only 20% cell invasion, whereas the MDA‐MB‐468 cells exhibited ~50% cell invasion at the same concentration (Figure 3). The MDA‐MB‐231 cells were more sensitive to LfcinB than the MDA‐MB‐468 cells. The numbers of invading MCF7 and SKBR3 cells were non‐significant. The normal MCF‐10A cells were non‐invasive (data not shown).

### 3.3 Impairment of tumour growth by LfcinB

**FIGURE 4:**
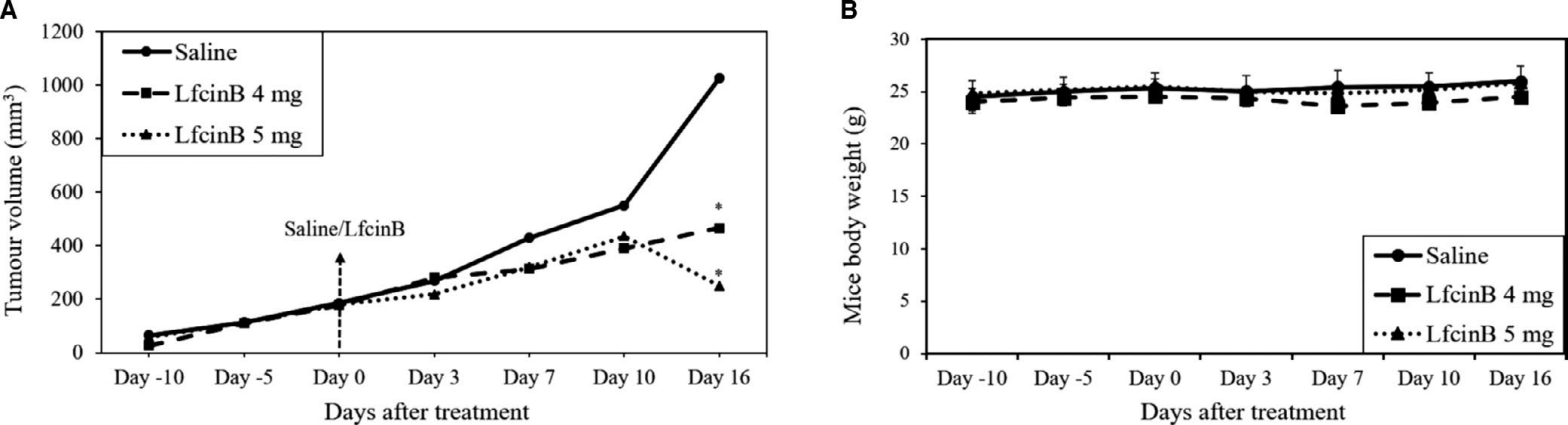
Intratumoural injection of LfcinB reduces tumour volume but not mice body weight. (A) Graph depicting the average tumour volume in mice that received intratumoural injections of saline or LfcinB (4 mg or 5 mg/mouse) monitored over time. (B) Graph depicting the average bodyweight of mice recorded over the corresponding period. *The result is significant at *P* < .05

The tumour growth rate was slower in mice that received intratumoural injections of LfcinB peptide compared to control mice treated with saline (Figure 4A). The difference in size was apparent from day 7 post‐injection, and over time, the gap between the treated and control groups (5 mice/group) became wider. By day 16, while tumour size in the control group reached ~1,000 mm^3^, that of the treatment group remained <500 mm^3^. Throughout the experimental period, the bodyweight of the mice was found to be stable in all the groups (Figure 4B).

**FIGURE 5:**
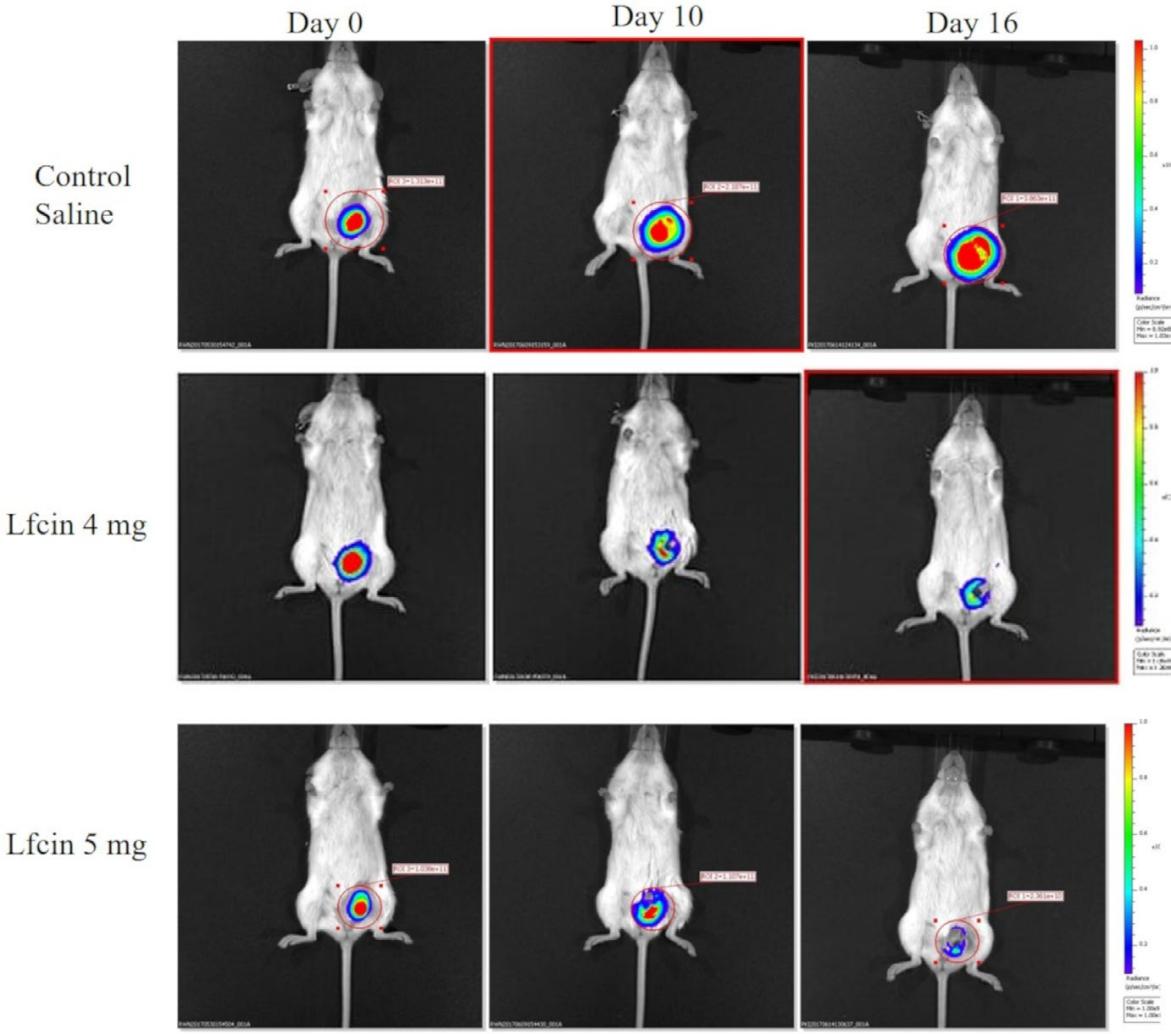
LfcinB reduces tumour size and density as depicted by live imaging of mice by IVIS. Photographs depicting tumour size and density taken using the in vivo imaging systems (IVIS, Perkin Elmer) to detect the luminescence of MDA‐MB‐231‐GFP‐luc cells in the tumours. Images were taken on the day of first injection (day 0) followed by day 10 and day 16 post‐treatment

Imaging of live mice using the IVIS^®^ Spectrum revealed that tumour size and density increased over time in the control group, as depicted by an increasingly intense red colour compared to the LfcinB‐treated groups that exhibited a decrease in tumour size, as well as in density. The LfcinB‐treated mice had smaller tumours with lower density, as indicated by the shift towards blue colour coupled with a decrease in red colour by day 16 (Figure 5).

**FIGURE 6:**
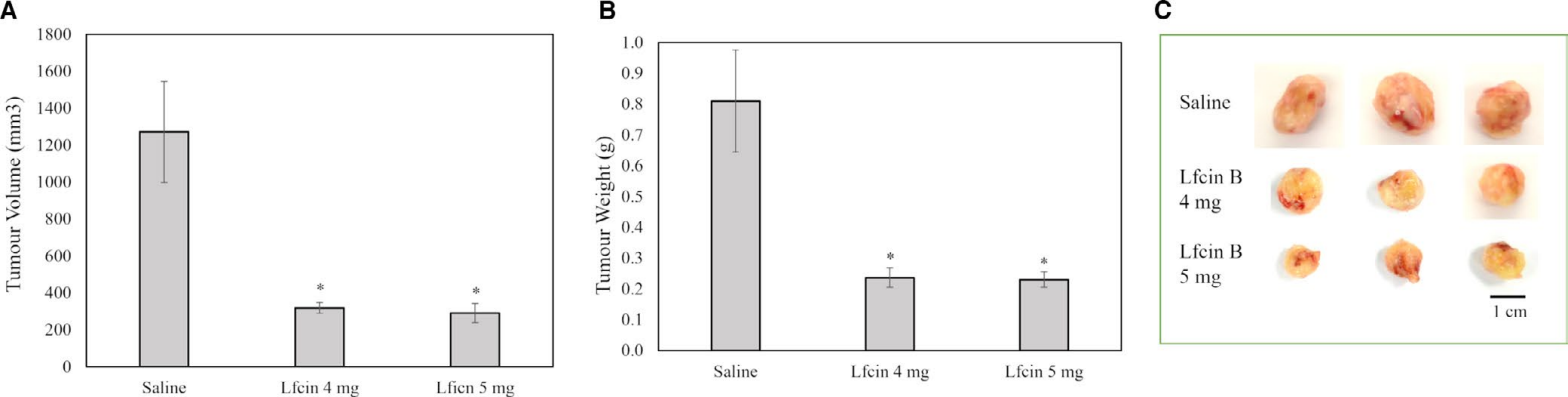
LfcinB reduces excised tumour volume and weight. Graphs depicting (A) the average volume and (B) the average weight of excised tumours from mice that received intratumoural injections of saline or LfcinB (4 or 5 mg/mouse). *The result is significant at *P* < .05. (C) Photographs of tumours excised from the different groups of mice

Excised tumour volume and weight exhibited the same decreasing trend. The control group had a mean tumour volume of 1,272 mm^3^, whereas the treatment groups had mean volumes of 319 and 291 mm^3^, respectively (Figure 6A). Similarly, tumour weight decreased from a mean of 0.81 g in the control group to ~0.23 g in the LfcinB‐treated groups (Figure 6B). Images of the excised tumours revealed a marked difference between the control and treated groups (Figure 6C).

### 3.4 Induction of apoptosis in tumours treated with LfcinB

**FIGURE 7:**
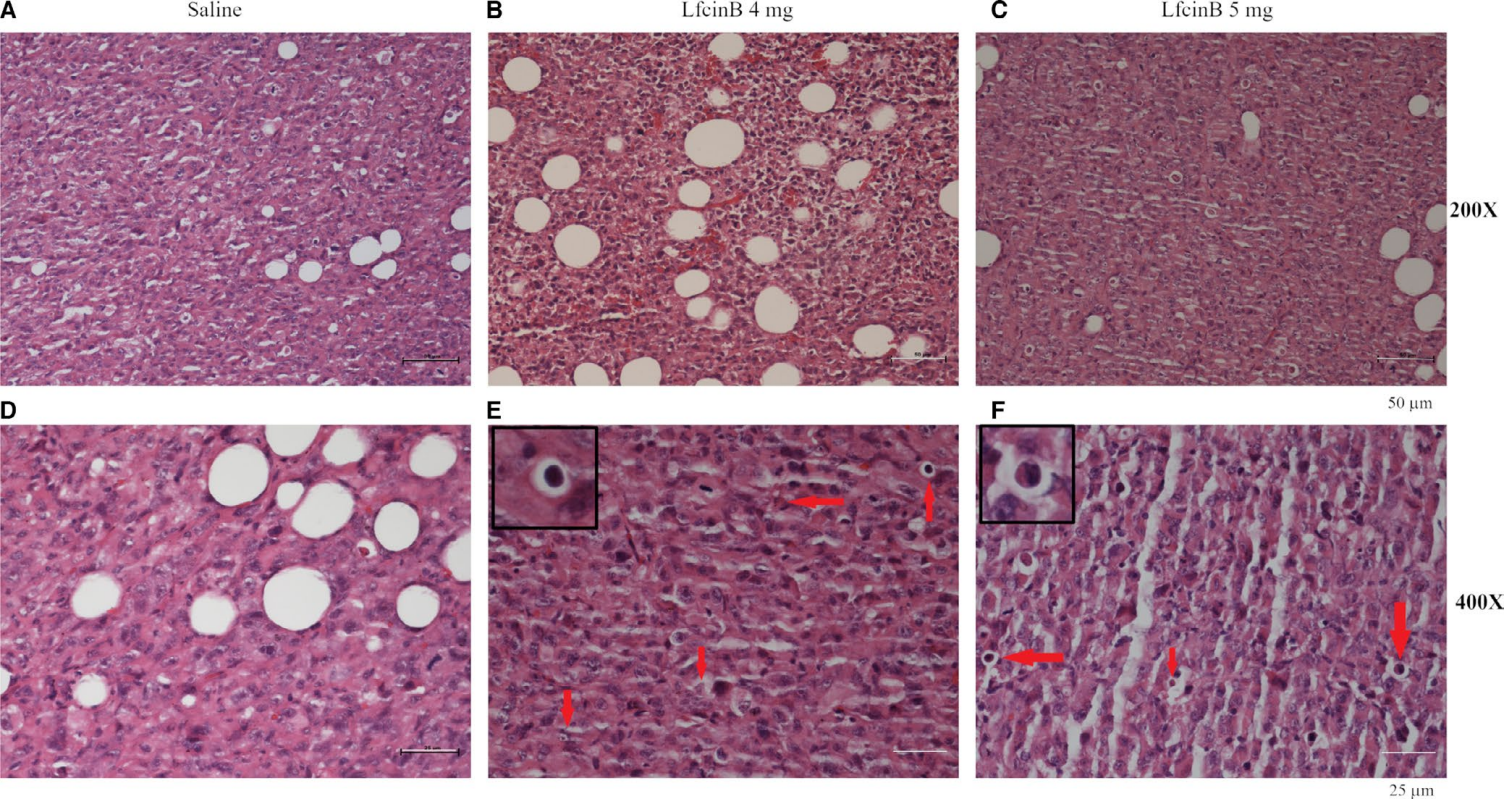
LfcinB induces apoptosis in excised tumour sections as depicted by histochemical analysis. Representative sections of tumours from mice treated with saline (A, D) or LfcinB 4 mg (B, E) or LfcinB 5 mg (C, F) shown at 200X (A, B, C) or 400X (D, E, F) magnification depicting apoptotic bodies marked by red arrows. Apoptotic cells are shown at higher magnification within E and F. Scale bars at 50 µm for (A, B, C) and at 25 µm for (D,E,F)

H&E staining of tumour sections from the LfcinB‐treated mice revealed an increased number of apoptotic cells compared to the control mice (Figure 7). This indicated that the intratumoural injection of LfcinB induced the apoptosis of the tumour cells, causing the tumours to shrink.

The current findings confirm the inhibitory effects of LfcinB on cancer cell lines in vitro as well as in vivo, and its ability to induce apoptosis and inhibit cancer cell invasion without affecting normal cells. These characteristics are the prerequisite for targeted therapy and indicate that LfcinB may be a valuable candidate for breast cancer treatment.

## 4 DISCUSSION

The development of peptides as anticancer agents is aimed at increasing the target specificity to cancer cells while decreasing toxicity and undesirable side effects. Anticancer peptides are generally cationic and exert their toxic effects by interacting with the anionic cancer cell membrane.^21^, ^22^ LfcinB is a cationic peptide rich in basic amino acids with a net positive charge of 7.84,^23^ and its selective targeting of cancer cells might be attributed to their elevated negative membrane potential.^22^ The differential sensitivity of breast cancer cell lines to therapeutic agents has been reported with the antimicrobial peptide Tempoprin‐1CEa, while a similar observation with bladder cancer cell lines was made with the peptide Magainin.^21^, ^24^ The MCF7 cell line is luminal A type and the SKBR3 cell line is Her2‐positive; the two triple‐negative cell lines, MDA‐MB‐468 and MDA‐MB‐231, are basal type and Claudin‐low type, respectively.^25^, ^26^ Chemotherapeutic drugs targeting Her2 have exhibited a sensitivity ranking in breast cancer cell lines as follows: SKBR3>MDA‐MB‐468>MCF7>MDA‐MB‐231.^27^ The SKBR3 cells, which lack functional p53, but have functional caspase‐3 were the most sensitive to the drugs used in the present study. In contrast, the MCF7 cells which have functional p53, but not functional caspase‐3, were relatively resistant. While the MCF7 cells are responsive to hormone therapy and are associated with the most favourable prognosis, they are relatively resistant to other drugs as well as LfcinB.^28^, ^29^

Smaller peptide derivatives of LfcinB have been synthesized and have exhibited anticancer properties. A study using the core peptide of LfcinB found that a net positive charge of +7 facilitated antitumour activity.^30^ The modification of the LfcinB peptide to cluster the cationic residues into a single region caused it to be 9.6‐fold more cytotoxic to the HL‐60 leukaemic cell line.^31^ Lactoferrin‐derived peptides have been reported to have weak cytotoxicity against MDA‐MB‐231 cells, while synthetic peptides in dimeric and tetrameric forms designed with the canonical motif RRWQWR of LfcinB are highly cytotoxic to the MDA‐MB‐468 and MDA‐MB‐231 breast cancer cell lines.^19^, ^20^ Even MCF7 cells, which are only moderately sensitive to LfcinB, have been found to be highly sensitive to the lactoferricin core peptide, RRWQWR.^32^ LfcinB causes no cytotoxicity to several normal cell lines tested,^14^, ^32^ has immunomodulatory and anti‐inflammatory properties^33^, ^34^ and does not elicit adverse side effects.

A variety of mechanisms have been proposed to underlie the anticancer effects of lactoferrin, LfcinB and its derivatives. A common mechanism proposed by many is the induction of apoptosis, membrane disruption and cell cycle arrest in cancer cells treated with lactoferrin or its derivatives.^35^, ^38^ Human lactoferrin was found to arrest the cell cycle at the G1 to S transition phase in MDA‐MB‐231 cells by inhibiting cyclin‐dependent kinases.^36^ In another study, lactoferrin was reported to induce cell cycle arrest and inhibit the mTOR signalling pathway, thereby inducing stress, but not apoptosis of breast cancer cell lines.^37^ Both lactoferrin and LfcinB were found to induce apoptosis by modulating the expression of pro‐apoptotic and anti‐apoptotic proteins and caspases in different cancer cell lines, as well as in implanted tumours in animal models.^13^, ^15^, ^16^, ^31^, ^34^, ^35^, ^36^ The induction of reactive oxygen species (ROS) was implicated by some studies in the apoptotic pathway induced by LfcinB.^11^, ^12^, ^40^ Mitochondrial death pathways and caspases have been proposed to be involved in apoptosis induced by LfcinB.^14^, ^31^, ^41^ JNK signalling pathway, which is implicated in mitochondria‐mediated apoptosis, was induced by LfcinB in oral cancer cells.^42^ Anti‐angiogenic properties have also been attributed to LfcinB as evident by its anti‐VEGF effect and decreased expression of matrix metalloproteases (MMPs).^43^

Both lactoferrin and lactoferricin have tumour‐suppressing functions. The expression of lactoferrin cDNA effectively reduced tumours derived from breast cancer as well as cervical cancer.^44^, ^45^ The direct tumour‐suppressive effects of LfcinB have been observed in in vivo studies using animal models.^39^, ^40^, ^41^, ^42^ In melanoma and colon carcinoma tumours, disruption of cell membrane, lysis and haemorrhagic necrosis was observed.^46^ Administration of LfcinB dendriplexes intravenously suppressed the growth of A431 and B16‐F10 tumour xenografts in mice and prevent tumour metastasis.^47^, ^49^ The intratumoural injection of a 9‐mer peptide, LTX‐302, which is a derivative of LfcinB, caused tumour necrosis and tumour infiltration by inflammatory cells followed by complete regression of tumours in a T lymphocyte–dependent manner in immunocompetent BALB/c mouse tumour model.^48^ LTX‐315, an oncolytic peptide derived from LfcinB, was shown to exert an additive effect when used along with chemotherapy as a combination therapy against triple‐negative breast cancers in a mouse model.^50^ It was also able to modulate immune response and reprogram the tumour microenvironment, and is being evaluated in clinical trials.^51^

Our study has shown that LfcinB is effective against different breast cancer cell lines including triple‐negative types and is capable of inducing apoptosis, and inhibiting cell invasion. Intratumoural administration of LfcinB caused significant reduction in tumour burden in vivo. Furthermore, it specifically targeted cancer cells without affecting the normal cells. These properties make LfcinB a promising natural product to be safely considered as a candidate for combination therapy against breast cancer. It can help minimize the dose of chemotherapeutic drugs thereby mitigating the side effects and reduce the overall cost of cancer treatment by serving as a cheaper alternative. Further studies are to be undertaken to evaluate this peptide for treatment of human breast cancer.

## CONFLICT OF INTEREST

The authors confirm that there is no conflict of interest.

## AUTHOR CONTRIBUTIONS

**Rizdwan Rahman:** Formal analysis (supporting); Investigation (lead). **Alyssa Danielle** **Fonseka:** Investigation (supporting). **Shiang‐Chia Sua** **:** Investigation (supporting). **Munirah Ahmad:** Methodology (supporting); Resources (supporting); Supervision (supporting); Writing‐review & editing (supporting). **Ramkumar Rajendran:** Methodology (supporting); Resources (supporting); Supervision (supporting); Writing‐review & editing (supporting). **Stephen** **Ambu:** Methodology (supporting); Resources (supporting); Supervision (supporting); Writing‐review & editing (supporting). **Fabian** **Davamani:** Conceptualization (supporting); Formal analysis (supporting); Methodology (supporting); Project administration (supporting); Resources (equal); Supervision (supporting); Writing‐review & editing (supporting). **Alan Soo‐Beng Khoo:** Methodology (supporting); Resources (supporting); Supervision (supporting); Writing‐review & editing (supporting). **Ebenezer** **Chitra:** Conceptualization (lead); Formal analysis (lead); Methodology (lead); Project administration (lead); Resources (lead); Supervision (lead); Writing‐original draft (lead); Writing‐review & editing (lead).

## Supporting information

Fig S1

## Data Availability

All data generated and analysed during this study are included in this article. Further details are available upon request.
