# Supplementary figures and images for: Inhibition of breast cancer xenografts in a mouse model and the induction of apoptosis in multiple breast cancer cell lines by lactoferricin B peptide
Source: J Cell Mol Med. 2021 Jul 8;25(15):7181–9. doi: 10.1111/jcmm.16748 (PMC8335703; doi:10.1111/jcmm.16748)

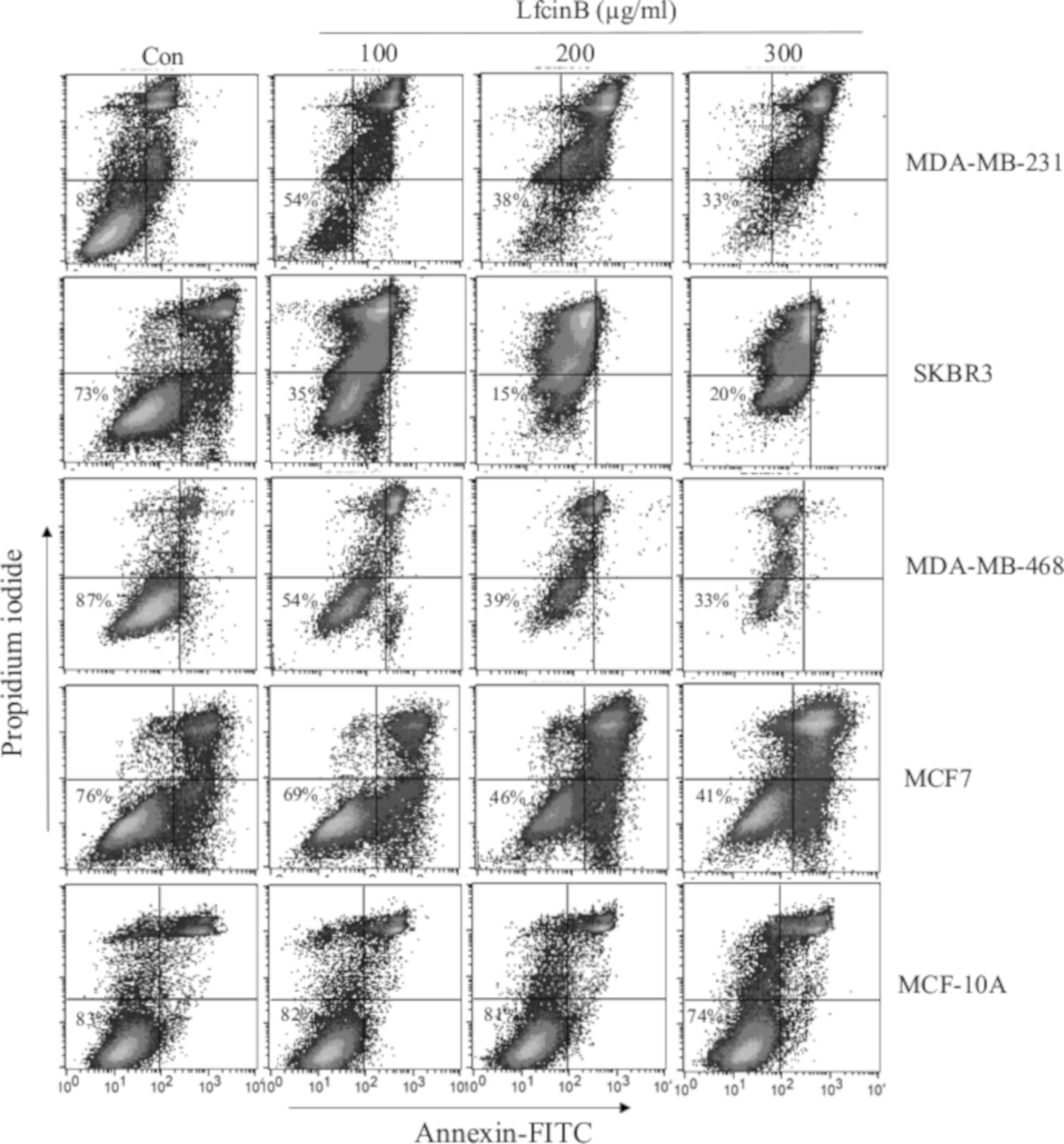

Supplement: Supplementary file 1 — Fig S1 [file JCMM-25-7181-s001.tif]
